# The Effect of Divergence in Feed Efficiency on the Intestinal Microbiota and the Intestinal Immune Response in Both Unchallenged and Lipopolysaccharide Challenged Ileal and Colonic Explants

**DOI:** 10.1371/journal.pone.0148145

**Published:** 2016-02-03

**Authors:** Stafford Vigors, John V. O’Doherty, Alan K. Kelly, Cormac J. O’Shea, Torres Sweeney

**Affiliations:** 1 School of Agriculture and Food Science, University College Dublin, Belfield, Dublin 4, Ireland; 2 School of Veterinary Medicine, University College Dublin, Belfield, Dublin 4, Ireland; 3 Faculty of Veterinary Science, University of Sydney, Sydney, Australia; Queen's University Belfast, UNITED KINGDOM

## Abstract

Feed efficiency is an important trait in pig production, with evidence to suggest that the efficiencies of a variety of biological systems contribute to variation in this trait. Little work has been conducted on the contribution of the intestinal innate immune response to divergence in feed efficiency. Hence, the objective of this study was to examine select bacterial populations and gene expression profiles of a range of targets relating to gut health and immunity in the intestine of pigs phenotypically divergent in feed efficiency in: a) the basal state; and (b) following an *ex-vivo* lipopolysaccharide (LPS) challenge of ileal and colonic tissue. Male pigs (initial BW 22.4 kg (SD = 2.03)) were fed a standard finishing diet for the final 43 days prior to slaughter to evaluate feed intake and growth for the purpose of calculating residual feed intake (RFI). On day 115, 16 animals (average weight 85 kg, SEM 2.8 kg), designated high RFI (HRFI) and low RFI (LRFI) were slaughtered. The LRFI pigs had increased *lactobacillus* spp. in the caecum compared to HRFI pigs (P < 0.05). RFI groups did not differ in the expression of the measured genes involved in the innate immune system in the basal ileal or colonic tissues (P > 0.10). Interestingly, there was an interaction between RFI and LPS for the cytokines *IL-8*, *IL-1*, *IL-6*, *TNF-α*, Interferon-γ (*IFN-γ*) and *SOCS3*, with the LRFI group having consistently lower gene expression in the colon following the LPS challenge, compared to the HRFI group. The lower gene expression of SOCS and cytokines following an *ex vivo* LPS challenge supports the theory that a possible energy saving mechanism exists in the intestinal innate immune response to an immune challenge in more feed efficient pigs.

## Introduction

Residual feed intake (RFI) is a useful trait in understanding the biological mechanisms that influence feed efficiency [1–3]. Maintaining a competent immune system and mounting effective immune responses is metabolically costly to the host, necessitating trade offs with other nutrient-demanding processes such as growth, reproduction and thermoregulation [4]. Previous research has demonstrated that feed efficient pigs have enhanced antimicrobial enzyme activity, lower serum IL-8, myeloperoxidase and endotoxin levels [2]. However, the relationship between feed efficiency and the intestinal innate immune response is not fully understood. The innate immune response is the initial non-specific acute phase response to an infection compared to the long term B- and T- cell mediated adaptive or acquired immune response. The innate immune response is considered more metabolically costly than the adaptive immune response and therefore it has been hypothesised that activation of the innate immune system could have measurable effects on feed efficiency [5]. Following an infectious challenge, the proportion of lysine directed to the immune system has been shown to increase from 2 to 9%, a fact which illustrates the nutritional cost of the immune response to infection [6].

The innate immune system possesses both physical and chemical barriers to infection and is vital in interacting with the microbiota in the intestinal lumen [7]. The intestinal microbiota profoundly impacts an array of physiological, developmental, nutritional and immunological processes of the host, and influences host health and performance and may significantly impact feed efficiency [8]. Pathogenic bacteria are inhibited from damaging the host through the continuous maintenance of a physical barrier. This barrier function is maintained through the dynamic turnover of the mucosal layer and maintenance of tight junction proteins between the epithelial cells. Mucins are glycosylated proteins which are secreted by goblet cells which in combination with commensal bacteria form a physical barrier between the underlying epithelial cells and the luminal contents [9]. In combination with the mucosal layer, tight junction proteins are important in preventing pathogen entry by limiting intercellular spaces [10].

Pathogen recognition receptors (PRR), which are located on epithelial cells, sense invading pathogens through the recognition of pathogen-associated molecular patterns (PAMPs) [11]. The recognition of PAMPs leads to the release of cytokines from mast cells particularly TNF-α and IL-1, which coordinate the recruitment, and activation of phagocytic cells such as neutrophils and macrophages to the site of infection [12, 13]. The proliferation of these cells occurs in concert with enhanced phagocytosis, increased cytokine production, activation of the complement pathway and the release of lysosomal enzymes to further enhance the hosts ability to fight infection [13]. While cytokines are an important component of the immune response, overproduction of pro-inflammatory cytokines can have a negative impact on the gastrointestinal tract (GIT) of the host, including impairing intestinal integrity and epithelial function and subsequently reduced feed efficiency [14]. Therefore the objective of this study was to examine select bacterial populations and the gene expression profiles of a range of targets relating to gut health and immunity in the intestine of pigs phenotypically divergent in feed efficiency in: a) the basal state; and (b) following an *ex-vivo* lipopolysaccharide (LPS) challenge of ileal and colonic tissue. The first hypothesis of this study is that there would be differences in specific bacterial populations and components of the innate immune system between high RFI (HRFI) and low RFI (LRFI) pigs in the basal state. The second hypothesis is LRFI pigs would have lower gene expression profiles for intestinal innate immune response genes following an lipopolysaccharide (LPS) challenge.

## Materials and Methods

All procedures described in this experiment were conducted under experimental licence from the Irish Department of Health in accordance with the Cruelty to Animals Act 1876 and the European Communities (Amendments of the Cruelty to Animals Act 1876) Regulations, 1994. This experiment was approved by the Animal Research Ethics Committee in University College Dublin (AREC-P-10-72-ODoherty)

### Animal management and sample collection

Eight pigs per RFI group were used from a previously established pig population described by Vigors *et al*. [15]. Blood samples (10 ml) were collected from the vena jugularis by puncture into vacutainers (Becton, Dickinson, Drogheda, Ireland) at two different timepoints (d 56 and d 115) to facilitate IL-8 quantification. Blood samples were collected from the same pigs at each time point. On collection, blood samples were immediately stored in ice-water and centrifuged at 1,500 x *g* at 4°C for 15 min. The plasma was then split into borosilicate glass scintilation vials and stored at -20°C until analysis. Plasma IL-8 samples were analysed using an enzyme linked immunosorbent assay (ELISA) (Cusabio Biotech Co., LTD, Suffolk, UK). On d 115, following a 3 hour fast the 16 pigs were humanely sacrificed by lethal injection with Pentobarbitone Sodium BP (Euthatal; Merial Animal Limited) at a rate of 0.71ml /2 kg body. Digesta for microbial and volatile fatty acid (VFA) analysis and tissue for gene expression analysis of a panel of genes encoding mucin, tight junction proteins, cytokines, toll-like receptors (TLR) and suppressor of cytokine signaling (SOCS) genes were then collected.

### Microbial & volatile fatty acid analysis

Immediately after slaughter, the entire digestive tract was removed by blunt dissection and digesta (approximately 10 (sd ± 1) g) was removed from the caecum and from the second loop of the ascending colon using sterile instruments and was stored in separate, sterile containers (Sarstedt, Wexford, Ireland) for further VFA and microbial analysis. Microbial genomic DNA was extracted from the faecal samples using a QIAamp DNA stool kit (Qiagen Inc., Valencia CA) in accordance with the manufacturer’s instructions. The quantity and quality of DNA were assessed using a Nanodrop apparatus (ND 1000, Thermo Fisher Scientific Inc., Waltham, MA). Standard curves from pooled aliquots of digesta microbial DNA and used to estimate absolute numbers of colonic *Enterobacteriaceae*, *Firmicutes*, *Bifidobacteria*, *Lactobacillus* and *Bacteroides* [16–18]. Standard curves were generated from a combined aliquot of feces and digesta from all pigs, which served as the source of bacterial genomic DNA. Pooled bacterial genomic DNA was extracted from the samples as described above. Species and genus specific primers (Table 1) were selected to amplify a segment of the gene encoding 16s rRNA using bacterial DNA from the pooled feces and digesta as a template using PCR. Thermal cycling conditions were as described for quantitative real-time PCR below. Standard curves were generated by quantitative real-time PCR of serial dilutions of these amplicons using the same genus and species-specific primers to permit absolute quantification based on gene copy number [16–18]. Genus- and species- specific primers (Table 1) were used for absolute quantification of select bacterial groups based on gene copy number in the faecal matter using qPCR on the ABI 7500 Real-Time PCR System (Applied Biosystems Limited, Carlsbad, Ca). For bacterial groups, QPCR was carried out in a final reaction volume of 20 μl containing 1 μl of template DNA, 1μl of forward and reverse primers (100 pM), 10 μl of SYBR Green PCR Master Mix (Applied Biosystems Limited, Carlsbad, Ca) and 8 μl of nuclease-free water. The thermal cycling conditions involved an initial denaturation step at 95°C for 10 min followed by 40 cycles of 95°C for 15s and 65°C for 1min. Dissociation analyses of the PCR products were carried out to confirm the specificity of the resulting PCR products. The mean threshold cycle values from the triplicate of each sample were used for calculations. The estimates of gene copy numbers for select bacteria were log-transformed, and they are presented as gene copy numbers/g faeces.

**Table 1:** Oligonucleotide sequences of forward and reverse primers used for QPCR of bacterial 16s rRNA.

Target bacteria	Forward primer (5’-3’) Reverse primer (5’-3’)	Amplicon size (bp)	T_m_ (°C)
*Firmicutes*	GGAGTATGTGGTTTAATTCGAAGCA	126	59
	AGCTGACGACAACCATGCAC		
*Bifidobacteria*	CGGGTGAGTAATGCGTGACC	125	59
	TGATAGGACGCGACCCCA		
*Lactobacillus*	TGGATCACCTCCTTTCTAAGGAAT	340	55
	TGTTCTCGGTTTCATTATGAAAAAATA		
*Enterobacteriaceae*	CATTGACGTTACCCGCAGAAGAAGC	190	58
	CTCTACGAGACTCAAGCTTGC		
*Bacteroides*	AACGCTAGCTACAGGCTT	276	54
	CAAATGTGGGGGACCTTC		

Digesta samples were collected from the colon and mixed with sodium benzoate and phenylmethylsulfonyl fluoride, to stop any bacterial activity and minimise the effects of post-thawing fermentation, which would influence final VFA concentrations. The VFA analysis was performed using GLC according to previous methods [19, 20].

### Ex-vivo tissue culture and LPS challenge

Colonic tissue was sampled from the same location as described for microbial samples. Ileal tissue was collected approximately 8cm from the ileo-caecal valve and tissue was dissected along the mesentary to remove digestive contents. Tissue sections of 1cm^2^, were cut from each tissue and stripped of the overlying smooth muscle. Two sections from each tissue were placed in 1ml of Dulbecco’s modified Eagle’s medium (Gibco, Invitrogen Corporation, Co. Dublin, Ireland), one in the presence of bacterial LPS (*Escherichia coli* strain B4; Sigma Aldrich Corporation, St. Louis, MO) at a concentration of 10 μg /ml. The other tissue sample was used as a control and incubated in sterile Dulbecco’s modified Eagle’s medium in the absence of LPS. Both challenged and unchallenged tissues were incubated at 37°C for 90 min before being removed, blotted dry and weighed. Approximately 1000–2000mg of the porcine ileal and colonic tissues were cut into small pieces and stored in 15ml RNAlater^™^ (Applied Biosystems Limited, Carlsbad, Ca) overnight at 48°C. RNAlater^™^ was then removed prior to storage at -80°C.

### RNA extraction and QPCR

Total RNA was extracted from ileal and colonic tissue samples (100 mg) using Trizol^™^ Reagent (Sigma-Aldrich, Arklow, Ireland) according to the manufacturer’s instructions. The crude RNA extract was further purified using the GenElute^™^ Mammalian Total RNA Miniprep Kit (Sigma-Aldrich, Arklow, Ireland) according to the manufacturer’s instructions, a DNase step was included (On-Column DNase I Digestion Set, Sigma-Aldrich, USA). The total RNA was quantified using a NanoDrop-ND1000 Spectrophotometer (Thermo Fisher Scientific Inc. MA, USA) and the purity was assessed by determining the ratio of the absorbance at 260 and 280 nm. All total RNA samples had acceptable 260/280 nm ratios. In addition, RNA integrity was established on the Agilent RNA 6000 Nanochip bioanalyser kit. Total RNA (1 μg) was reverse transcribed (RT) using First Strand cDNA Synthesis Kit, (Applied Biosystems Limited, Carlsbad, Ca) using oligo dT primers in a final reaction volume of 20 μL, according to the manufacturer’s instructions. The cDNA was then adjusted to a volume of 120μl using nuclease free water. The relative expression of select genes was analysed by QPCR using the ABI Prism 7500 FAST sequence detection system (Applied Biosystems Limited, Carlsbad, Ca). All primer pairs are listed in Table 2. All reactions were performed in duplicate in a total volume of 20 μL containing 10 μL of SYBR Green PCR Mastermix, (Applied Biosystems Limited, Carlsbad, Ca) 1.0 μL forward and reverse primer (300 pM each)), 8 μL of RNAse free water, and 1 μL of template cDNA (5.0 ng of RNA equivalents). The 2-step PCR program was as follows: 95°C for 10 min for 1 cycle, followed by 95°C for 15 sec and 60°C for 1 min for 40 cycles. Minus reverse transcriptase and no template controls were included. Primers were designed for each gene of interest (Primer Express Software v2.0, Applied Biosystems Limited, Carlsbad, Ca) and the specificity of all primers was confirmed by dissociation analysis. All assays had efficiency between 90 and 110%. The most optimally stable reference targets were identified using the geNorm application within the qbase^PLUS^ software package [21] (Biogazelle, Zwijnaarde, Belgium) and confirmed for this study (V < 0.15). The normalisation factor was calculated using the qbase^PLUS^ algorithm from the reference targets *YWHAZ* and *ACTB*. Normalised relative quantities (CNRQ) for each target was used in subsequent statistical analysis.

**Table 2:** Porcine oligonucleotide primers used for real-time PCR.

Gene	Accession Number	Forward primer (5’-3’) Reverse primer (5’-3’)	Melting Temp (°C)	Product Length (BP)
**Reference targets**				
*B2M*	NM_213978.1	CGGAAAGCCAAATTACCTGAAC	58.2	83
		TCTCCCCGTTTTTCAGCAAAT	58.4	
*ACTB*	AY550069.1	CAAATGCTTCTAGGCGGACTGT	60.9	75
		TCTCATTTTCTGCGCAAGTTAGG	59.5	
*GAPDH*	AF017079.1	CAGCAATGCCTCCTGTACCA	60.0	72
		ACGATGCCGAAGTTGTCATG	58.9	
*YWHAZ*	XM_001927228.1	GGACATCGGATACCCAAGGA	58.5	71
		AAGTTGGAAGGCCGGTTAATTT	58.7	
**Cytokines**				
*IL-1*^a^	NM_214029.1	CAGCCAACGGGAAGATTCTG	63.0	76
		ATGGCTTCCAGGTCGTCAT	60.9	
*IL-6*	AB194100	AGACAAAGCCACCACCCCTAA	55.2	69
		CTCGTTCTGTGACTGCAGCTTATC	59.9	
*IL-8*	NM_213867.1	GCACTTACTCTTGCCAGAACTG	61.9	82
		CAAACTGGCTGTTGCCTTCTT	61.7	
*IL-10*	NM_214041.1	GCCTTCGGCCCAGTGAA	63.4	71
		AGAGACCCGGTCAGCAACAA	63.1	
*TNF-α*	NM_214022.1	TGGCCCCTTGAGCATCA	62.8	68
		CGGGCTTATCTGAGGTTTGAGA		
*IFN- γ*	NM_213948.1	TCTAACCTAAGAAAGCGGAAGAGAA	61.1	81
		TTGCAGGCAGGATGACAATTA	61.5	
**SOCS**				
*SOCS1*	EW101597	CGCCCTCAGTGTGAAGATGG	62.0	110
		GCTCGAAGAGGCAGTCGAAG	61.4	
*SOCS2*	NM 001097461	CTGCGCATCGAATACCAAG	58.0	190
		TGTAGAGCGGTTTGGTCAG	56.9	
*SOCS3*	NM 001123196	CACTCTCCAGCATCTCTGTC	62.0	105
		TCGTACTGGTCCAGGAACTC	63.5	
*SOCS4*	ES445034	TCCTGGGACAGGCTCTATG	59.0	170
		GGTACTTGGGAGGTGTTTC	60.1	
*SOCS5*	DB784235	ACGCTGTGTTTGCAGTCTC	58.0	89
		ACTTTCCAAGCTCCCTGTC	58.9	
*SOCS6*	XM 001926570	ATCTCTAGCCGGTGACTTCG	62.0	178
		GCCCTTCTGCTTCTGTTTCG	60.1	
*SOCS7*	ENSSSCT00000019652	CACTTGTGGACGTGGACATC	62.0	162
		GGAAAGACTGCAGGGAAGAC	55.5	
**Toll-like receptors**				
*TLR-2*	XM_005653579.1	GGCAAGTGGATTATTGACAACATC	58.6	94
		ACCACTCGCTCTTCACAAAGTTC	61.2	
*TLR-4*	NM_001293317.1	TGCATGGAGCTGAATTTCTACAA	58.6	140
		GATAAATCCAGCACCTGCAGTTC	59.9	
*TLR-6*	NC_010450.3	TCTGCTCAAGGACTTCCGTGTA	61.0	85
		CAATGCCAGCCCAGTGACT	60.3	
**Mucins**				
*MUC2*	AK231524	CAACGGCCTCTCCTTCTCTGT	63.1	70
		GCCACACTGGCCCTTTGT	62.1	
*MUC4*	XM_001926442.1	GATGCCCTGGCCACAGAA	63.3	89
		TGATTCAAGGTAGCATTCATTTGC	62.4	
*MUC5*	AF054584	CCCCTCGTCTCCTTTTACC	62.1	71
		GGATGTCGCCAGAGACTGAGTA	61.7	
*MUC12*	FJ715471	GACTAACAAGAATTTCACAAAAGAGCTAA	60.8	87
		GCCATCTGAGTCTTGAACTTTTGA	61.9	
*MUC20*	NM_001113440	AGGCAGTTACAACATCCACAGAAG	61.8	82
		CTGTAGACCATGGCCGAGAAC		
**Tight junctions**				
*ZO1*	XM_005659811.1	TGAGAGCCAACCATGTCTTGAA	59.9	76
		CTCAGACCCGGCTCTCTGTCT	60.0	
*CLDN2*	NM_001161638.1	AGGCCTCCTGGGCTTCAT	60.2	64
		GGAGTAGAAGTCCCGCAGGAT	61.0	

^a^ *IL*: interleukin; *TNF-α*: tumour necrosis factor alpha; *IFN-γ*: interferon gamma; *SOCS*: suppressor of cytokine signalling; *MUC*: mucin; *ZO1*: tight junction protein 1; *CLDN2*: claudin2

### Statistical analysis

The data were initially checked for normality using the UNIVARIATE procedure of SAS. Bacterial data were analyzed using the MIXED procedure as a complete randomised design with RFI the main effect, sow as a random effect and the pig was the experimental unit. Gene expression data in both the ileum and colon was analysed as a 2 x 2 factorial arrangement using the MIXED procedure of SAS with the pig as the experimental unit. For the gene expression data the statistical model used included the effects of RFI and challenge (LPS or Basal) and their associated two-way interactions with sow included as a random effect. All data presented in the tables are expressed as least squares means ± standard error of the mean (SEM). Means were separated using the Tukey-Kramer method. The probability value, which denotes statistical significance, was *P* < 0.05.

## Results

### Performance and feed efficiency

Differences in feed intake, performance and feed efficiency traits are presented in Table 3. The LRFI group had a lower RFI value (-0.14) compared to both medium (0) and HRFI (0.19) pigs (P < 0.001). The LRFI pigs had lower average daily feed intake (ADFI) than pigs ranked as MRFI or HRFI (P < 0.001). On average the LRFI group consumed 128g and 330g less feed than their counterparts ranked either MRFI or HRFI, respectively. The LRFI pigs had a lower feed conversion ratio (FCR) than pigs ranked as MRFI or HRFI (P < 0.001). The mid-test metabolic body weight (MBW), average daily gain (ADG) and final body weight (BW) did not differ between RFI groups (P > 0.05).

**Table 3:** Characterisation of average daily gain, intake and feed efficiency across all animals (n = 72) (Least square means and SEM).

RFI	High^e^	Medium	Low	SEM	*P*-value
No. Of Animals	18	27	27		
RFI^d^	0.19^a^	0.0^b^	-0.14^c^	0.08	**0.001**
ADFI	2.34^a^	2.13^b^	2.00^c^	0.04	**0.001**
FCR	2.36^a^	2.18^b^	2.03^c^	0.02	**0.001**
MBW	11.02	10.94	10.86	0.29	0.956
ADG	0.99	0.98	0.99	0.02	0.874
BW	77.70	76.68	76.62	1.36	0.833

^a, b, c^ Figures with different superscript within the row are significantly different.

^d^ RFI: Residual feed intake (Calculated d105); ADFI: Average daily feed intake (d56-d105); FCR: Feed conversion ratio; MBW: Mid-test metabolic body weight (BW^0.60^ measured d77); ADG: Average daily gain; BW: Body weight.

^e^ High = RFI was >0.5 SD above the mean; medium = RFI was ±0.5 SD above and below the mean; low = RFI was <−0.5 SD below the mean.

### Intestinal microbiota

The LRFI pigs had increased *Lactobacilli* numbers in the caecum compared to HRFI pigs (P > 0.05, Table 4). There was no difference between RFI groups for numbers of *Firmicutes*, *Bifidobacteria*, *Enterobacteria* or *Bacteroides* in either the caecum or colon (P > 0.10).

**Table 4:** Characterisation of the effect of residual feed intake on the abundance of selected gastrointestinal microbial groups present in ceacal and colonic digesta (gene copy number/g digesta) (Least square means and SEM).

RFI^a^^d^	High^b^	Low	SEM	*P*-value
**Caecum**^c^				
*Lactobacillus*	7.54	8.42	0.26	**0.040**
*Enterobacteriaceae*	10.26	9.03	0.95	0.377
*Bifidobacteria*	3.95	4.29	0.47	0.622
**Colon**				
*Lactobacillus*	8.27	8.65	0.22	0.245
*Enterobacteriaceae*	8.33	7.94	0.50	0.591
*Bifidobacteria*	5.01	5.10	0.18	0.733
*Bacteroides*	10.37	10.37	0.06	0.931
*Firmicutes*	13.36	13.46	0.09	0.436

^a^ RFI: Residual feed intake;

^b^ High = RFI was >0.5 SD above the mean; medium = RFI was ±0.5 SD above and below the mean; low = RFI was <−0.5 SD below the mean.

^c^ Digesta samples collected post slaughter d105

^d^ Eight pigs per RFI group

### Volatile fatty acids

The LRFI pigs tended to have increased total volatile fatty acid production in the caecum compared to the HRFI pigs (P < 0.10, Table 5). The LRFI pigs also tended to have increased molar proportions of butyric acid compared to the HRFI group.

**Table 5:** Characterisation of the effect of residual feed intake on the total volatile fatty acid (VFA) concentration and molar proportions in the caecum (Least square means and SEM).

RFI^a^	High^b^	Low	SEM	*P*-value
**Caecum**^c^^d^				
Total VFA (mmol/g digesta)	23.22	33.81	3.792	**0.072**
**Molar proportions**				
Acetic	0.84	0.82	0.02	0.382
Propionic	0.05	0.04	0.01	0.621
Butyric	0.07	0.10	0.01	**0.078**
Valeric	0.02	0.02	0.00	0.869
Iso-Butyric	0.02	0.03	0.01	0.424

^a^ RFI: Residual feed intake;

^b^ High = RFI was >0.5 SD above the mean; medium = RFI was ±0.5 SD above and below the mean; low = RFI was <−0.5 SD below the mean.

^c^ Digesta samples collected post slaughter d105

^d^ Eight pigs per RFI group

### Serum IL-8

The RFI groups did not differ in serum IL-8 levels on either day 56 or day 115 with IL-8 levels below the lower limits of detection by the assay (P > 0.05, Data not presented).

### Ileal tissue

There was no effect of RFI group on the gene expression of the measured mucin genes, TLRs, tight junction proteins, cytokines or SOCS genes in the basal ileum (P < 0.10).

**Fig 1:**
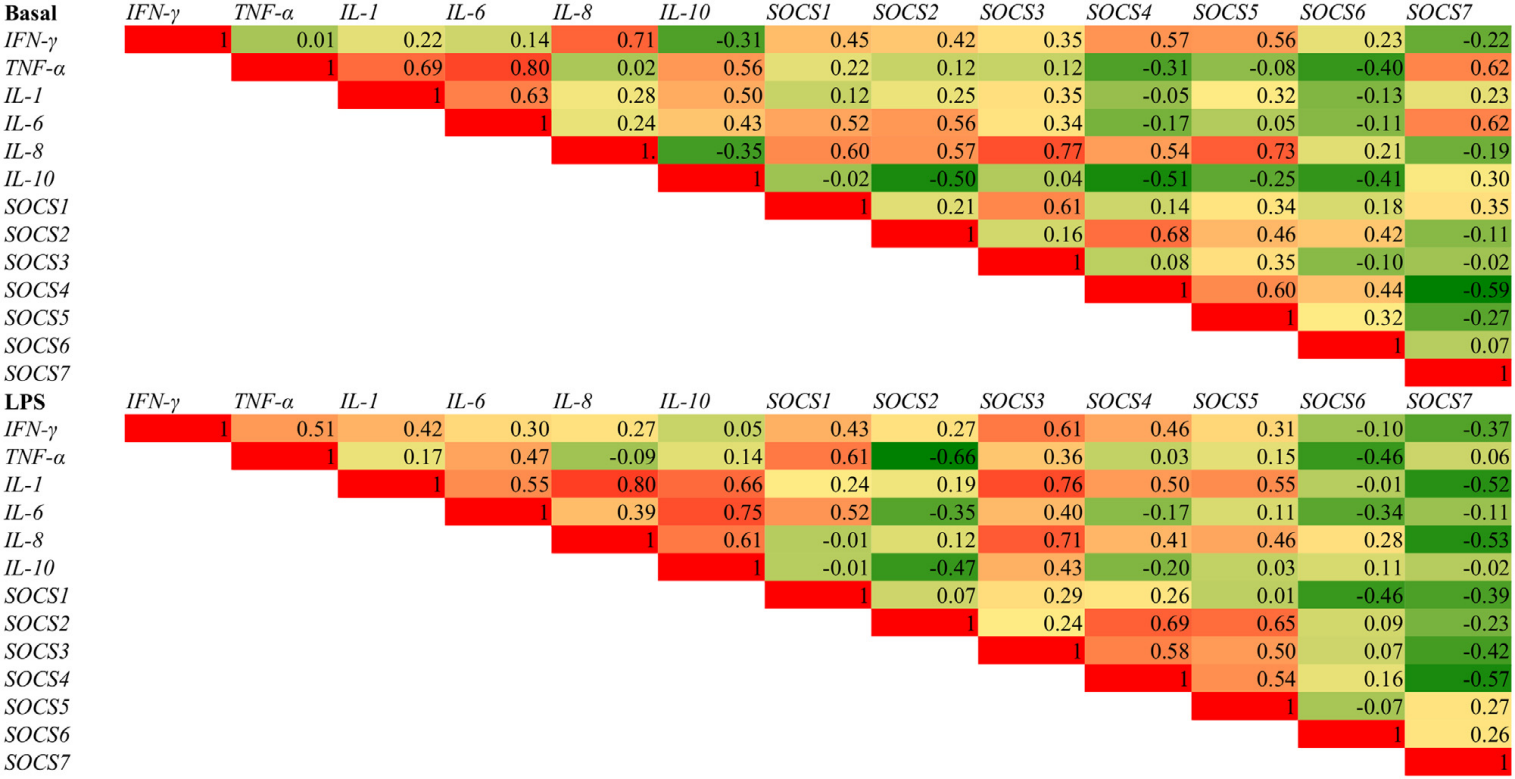
Correlation analysis between cytokines and SOCs genes in ileal tissue in the basal and LPS challenged tissue.

Following the LPS challenge the expression of *IL-1* (P < 0.01), *IL-6* (P < 0.01), *IL-8* (P < 0.05) *IL-10* (P < 0.01), and *IFN-γ* (P < 0.01) was increased compared to the unchallenged tissue (Table 6). LPS decreased the expression of *SOCS4* (P < 0.05) and *SOCS5* (P < 0.01) while it increased the expression of *SOCS1* (P < 0.05) and *SOCS3* (P < 0.05). The heat map representing the correlations (R^2^) between the cytokines and SOCS genes are presented in Fig 1. The LPS challenge reduced the expression of *TLR-2*, *TLR-4* and *TLR-6* (P < 0.05). LPS significantly down-regulated the expression of *MUC2* (P < 0.001), *MUC4* (P < 0.05) and *MUC20* (P < 0.05). The LPS challenge also reduced the expression of the tight junction proteins *ZO1* (P < 0.01) and *CLDN2* (P < 0.05).

**Table 6:** Characterisation of the gene expression of cytokines, suppressor of cytokine signalling genes (SOCS), toll-like receptor, mucins and tight junctions in both basal and lipopolysaccharide (LPS) challenged ileal tissue (Least square means and SEM).

Treatment	Basal	LPS^c^	SEM	*P*-value
**Cytokines**^b^				
*IFN-γ*^a^	0.93	1.41	0.17	**0.008**
*TNF-α*	0.94	1.33	0.21	**0.072**
*IL-1*	0.70	2.10	0.38	**0.001**
*IL-6*	0.75	1.72	0.28	**0.001**
*IL-8*	1.02	1.64	0.31	**0.046**
*IL-10*	0.65	1.77	0.27	**0.001**
**SOCS**				
*SOCS1*	0.83	1.46	0.24	**0.016**
*SOCS2*	1.36	1.07	0.25	0.247
*SOCS3*	0.95	1.38	0.17	**0.014**
*SOCS4*	1.17	0.98	0.09	**0.041**
*SOCS5*	1.18	0.98	0.07	**0.007**
*SOCS6*	1.12	1.01	0.11	0.282
**Toll-like receptors**				
*TLR2*	1.44	1.03	0.21	**0.046**
*TLR4*	1.27	1.01	0.11	**0.027**
*TLR6*	1.16	0.96	0.09	**0.039**
**Mucins**				
*MUC1*	1.26	1.19	0.20	0.465
*MUC2*	1.72	0.95	0.23	**0.002**
*MUC4*	1.76	1.04	0.32	**0.035**
*MUC20*	1.54	1.03	0.22	**0.027**
**Tight junctions**				
*ZO1*	1.21	0.96	0.07	**0.003**
*CLDN2*	1.79	1.12	0.32	**0.054**

^a^ *IFN- γ*: interferon gamma; *TNF-α*: tumour necrosis factor alpha; *IL*: interleukin; *SOCS*: suppressor of cytokine signalling; *MUC*: mucin; *ZO1*: tight junction protein 1; *CLDN2*: claudin2

^b^ Tissue samples collected post slaughter d105

^c^ Bacterial LPS *Escherichia coli* strain B4 at a concentration of 10 μg /ml for 3 hrs

There was no interaction between RFI*LPS on the gene expression of the measured mucin genes, TLRs, tight junction proteins, cytokines or SOCS genes.

### Colonic tissue

In the basal colon, RFI had no effect on the gene expression of the measured mucin genes, TLRs, tight junction proteins, cytokines or SOCS genes (P > 0.10).

**Fig 2:**
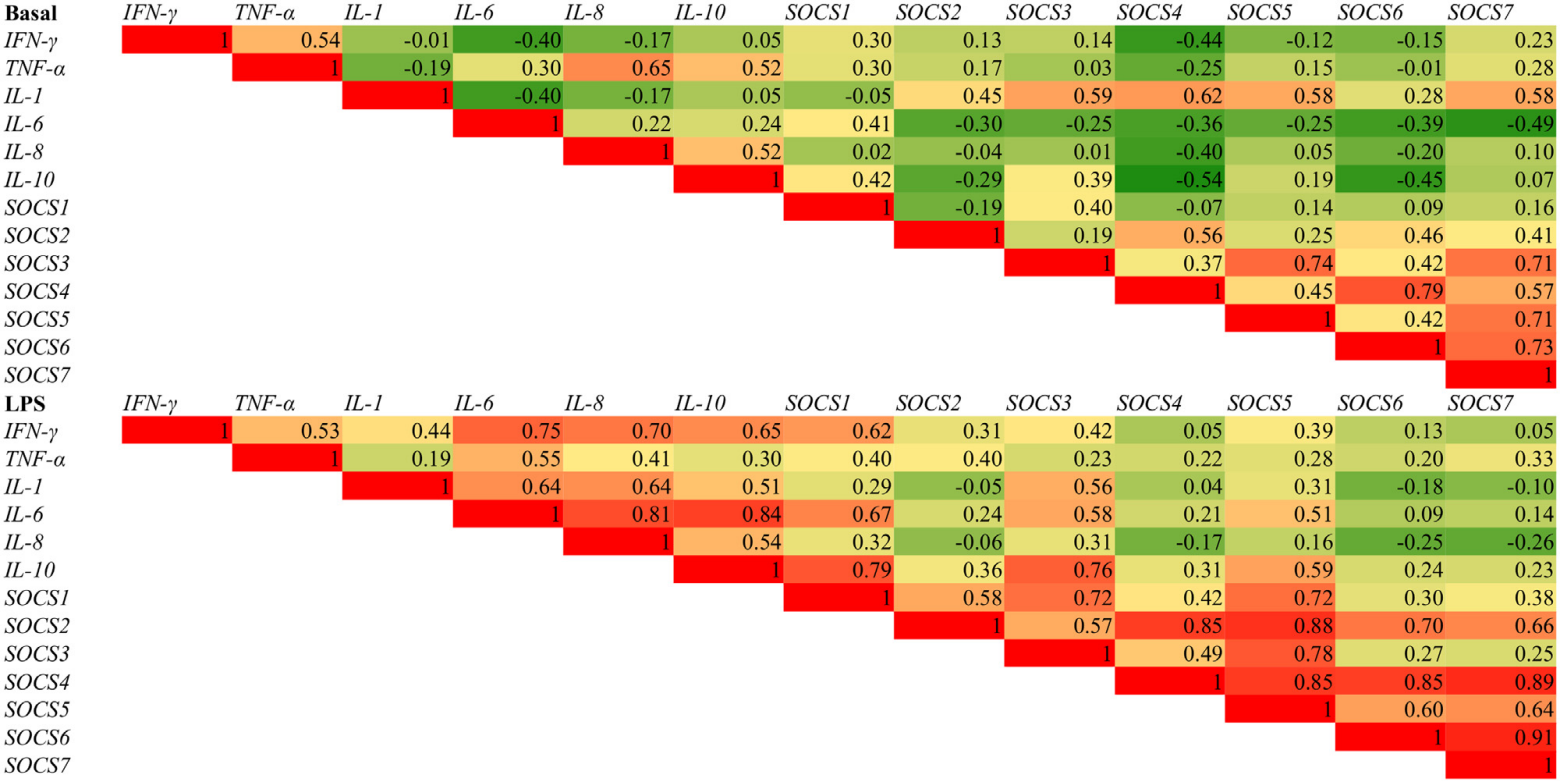
Correlation analysis between cytokines and SOCs genes in colon tissue in the basal and LPS challenged tissue.

The LPS challenge increased the expression of *IL-1* (P < 0.001), *IL-6* (P < 0.001), *IL-8* (P < 0.05), *IL-10* (P < 0.05), IFN- γ (P < 0.05) and *TNF-α* (P < 0.0001) (Table 7). The expression of *SOCS1* (P < 0.001) and *SOCS3* (P < 0.01) was increased following the addition of LPS while there was also a tendency towards decreased *SOCS6* (P < 0.10). The heat map (Fig 2) illustrates the interrelationships between cytokines and SOCS genes in the basal state and following the addition of LPS. Following the LPS challenge the expression of *MUC12* was reduced (*P* < 0.01). The expression of the measured TLRs and tight junction proteins were unaffected following the addition of LPS (P > 0.10).

**Table 7:** Characterisation of the gene expression of cytokines, suppressor of cytokine signalling genes, toll-like receptor, mucins and tight junctions in both basal and lipopolysaccharide (LPS) challenged colonic tissue (Least square means and SEM).

Treatment	Basal	LPS^c^	SEM	*P*-value
**Cytokines**^b^				
*IFN-γ*^a^	0.89	1.26	0.24	**0.011**
*TNF-α*	0.43	1.79	0.21	**0.001**
*IL-1*	0.51	1.62	0.18	**0.001**
*IL-6*	0.53	1.63	0.17	**0.001**
*IL-8*	0.62	1.38	0.27	**0.048**
*IL-10*	0.93	1.43	0.21	**0.021**
**SOCS**				
*SOCS1*	1.71	0.76	0.25	**0.001**
*SOCS2*	1.11	1.08	0.13	0.798
*SOCS3*	0.72	1.32	0.17	**0.001**
*SOCS4*	1.10	1.05	0.11	0.707
*SOCS5*	1.02	1.04	0.10	0.823
*SOCS6*	1.22	0.98	0.13	**0.081**
*SOCS7*	1.16	1.07	0.15	0.734
**Toll-like receptors**				
*TLR2*	1.21	1.05	0.19	0.401
*TLR4*	1.25	1.18	0.24	0.232
*TLR6*	1.23	1.04	0.19	0.335
**Mucins**				
*MUC2*	1.19	1.05	0.19	0.437
*MUC4*	1.04	1.05	0.15	0.977
*MUC5*	1.20	1.63	0.50	0.389
*MUC12*	1.44	0.90	0.18	**0.005**
*MUC20*	1.05	1.09	0.17	0.845
**Tight junctions**				
*ZO1*	1.21	1.13	0.17	0.425
*CLDN2*	1.79	1.19	0.19	0.183

^a^ *IFN- γ*: interferon gamma; *TNF-α*: tumour necrosis factor alpha; *IL*: interleukin; *SOCS*: suppressor of cytokine signalling; *MUC*: mucin; *ZO1*: tight junction protein 1; *CLDN2*: claudin2

^b^ Tissue samples collected post slaughter d105

^c^ Bacterial LPS *Escherichia coli* strain B4 at a concentration of 10 μg /ml for 3 hrs

**Fig 3:**
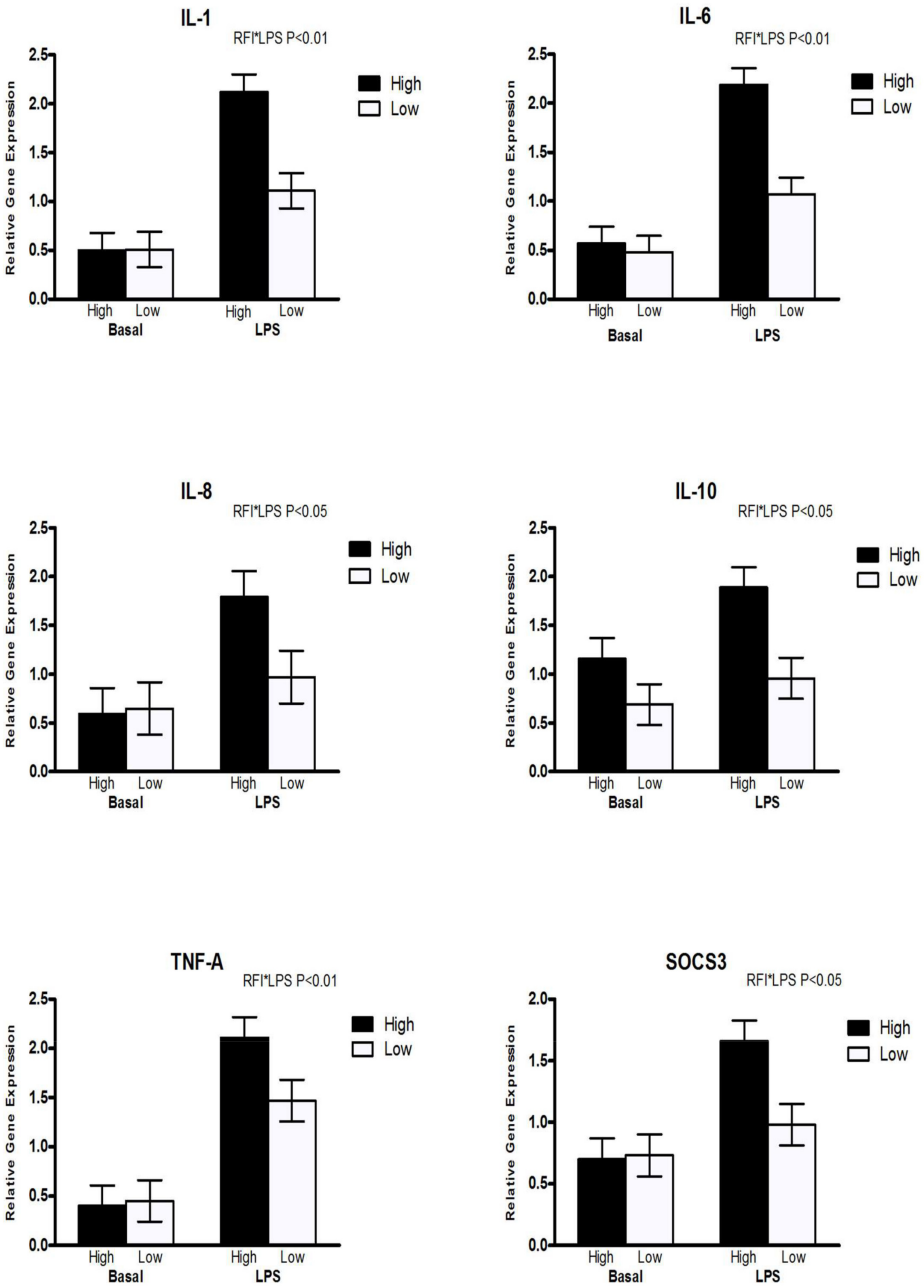
The interaction between residual feed intake and LPS in ex-vivo LPS challenged colonic tissue illustrating the higher expression of cytokines and SOCS in high RFI pigs compared to low RFI.

There was an interaction between RFI and LPS for the cytokines *IL-1* (P < 0.01), *IL-6* (P < 0.05), *IL-8* (P < 0.05), *IL-10* (P < 0.05) and *TNF-α* (P < 0.05, Fig 3) with HRFI pigs having a greater response to the LPS challenge compared to LRFI pigs. Similarly there was an interaction between RFI and LPS for *SOCS3* (P < 0.05) with HRFI pigs having a greater response to LPS than LRFI pigs. There was no interaction between RFI and LPS on the expression of mucins, TLRs or tight junctions (P > 0.10).

## Discussion

The hypothesis that specific bacterial populations and components of the innate immune system would differ between HRFI and LRFI pigs in the basal state was supported in part in this study. In the basal state LRFI pigs had increased *lactobacilli* numbers in the caecum, however there were no differences between the RFI groups with respect to differential gene expression of components of the innate immune response, including; mucins, toll-like receptors, tight junction proteins, cytokines, SOCS, or plasma IL-8 levels. This suggests that a heightened immune response is not a significant influence on the lower feed efficiency observed in HRFI pigs. The hypothesis that LRFI pigs would have lower gene expression profiles of immune response genes following an LPS challenge was supported in this study. In the LPS challenged tissue LRFI pigs had lower expression of the cytokines *IL-1*, *IL-6*, *IL-8*, *IL-10*, *TNF-α* and *SOCS3* following the challenge, suggesting a differential innate immune response to a challenge in pigs that differ in feed efficiency.

The LRFI pigs had increased *lactobacillus spp*. in the caecum compared to HRFI pigs. *Lactobacillus* spp. increases gut function and health [22]. Increased *lactobacilli* are associated with increased VFAs known to stimulate cell proliferation, differentiation and improve intestinal health [23, 24]. Lactic acid produced from *lactobacillus* spp. is reported to interact with the gut mucosal surface resulting in immunomodulatary effects [25]. Hence, the increase in *lactobacillus* spp. could be interpreted as an improvement to overall gut health in the LRFI group relative to the HRFI group. It may also be noteworthy to state that the log change in *Enterobacteriaceae* may also be impacting intestinal health. *Enterobacteriaceae*, especially *Escherichia coli* strains have been implicated in reductions in intestinal health with increases in diarrhea in pigs [26]. While nutrient absorption across the intestinal wall is the dominant method of nutrient utilisation, the intestinal microflora have an important metabolic function in the fermentation of non-digestible carbohydrates into a final product of VFAs, which can subsequently be used as an energy source. The VFAs (acetate, propionate and butyrate) stimulate epithelial cell proliferation and differentiation in the colon [24]. In this study LRFI pigs tended to have increased total VFA production in the caecum with a tendency towards increased molar proportions of butyric acid. Butyrate plays an important role in cell proliferation and development, suggesting that the increased molar proportions of butyric acid in the caecum in LRFI pigs may be having a positive impact on gut health [27, 28].

In the basal state, RFI groups did not differ in the expression of innate immune system related genes in either the ileum or colon, including mucins, TLRs, tight junctions, cytokines or SOCS. This is supported by the fact that RFI groups did not differ in circulating IL-8 levels. The lack of effect in both the *ex-vivo* tissues and in serum suggests that the RFI groups did not differ in their baseline innate immune response and hence in this instance does not contribute to the differences in feed efficiency [29].

To mimic the immediate innate immue response to a bacterial challange, ileal and colonic tissues were incubated with LPS *ex-vivo*. Lipopolysaccharide is a component of the outer membrane of gram-negative bacteria which binds the CD14/TLR4/MD2 receptor complex in many cell types including monocytes, dendritic cells, macrophages and B cells, subsequently promoting the secretion of pro-inflammatory cytokines [30]. There was an interaction between RFI and LPS in the colon with LRFI pigs having a lower response to LPS with regard to the relative gene expression of *IL-1*, *IL-6*, *IL-8*, *IL-10*, and *TNF-α*, relative to the HRFI pigs. There are a number of ways these findings could be interpreted. Cytokines block pathogen replication and are also involved in recruitment and activation of immune cells [13]. Therefore, the increased expression of a number of pro-inflammatory cytokines in response to LPS in HRFI pigs compared to LRFI pigs, suggests that these animals retain a capacity to respond more adequately to infection. The lower expression of cytokines in the LRFI group following the addition of LPS may therefore be inadequate to suppress the infection. However, Dunkelberger *et al*. [31] found LRFI pigs had greater ADG following infection by porcine reproductive and respiratory syndrome, suggesting that the reduced cytokine gene expression in the current study may not be detrimental to the health of the pig. Previously, Cotter and Van Eerden [32] concluded that the immune response of more feed efficient birds was also more effective in responding to infections, with a more focused partitioning of resources. The study found that efficient birds produced antibodies only in response to a *Salmonella Enteritidis* infection whereas inefficient birds produced antibodies in response to heat-killed *Salmonella Enteritidis*, which does not have capacity to cause disease. The results from the Cotter and Van Eerden [32] study suggest that the lower cytokine gene expression in the LRFI group in the current study is possibly a similar energy saving mechanism. This is further backed up through work by Mani *et al*. [2] where LRFI pigs had lower expression of inflammatory markers, IL-8 and myeloperoxidase, decreased serum endotoxin and enhanced activities of antimicrobial enzymes suggesting inefficient pigs expend greater energy fuelling their basal immune response, which subsequently has a negative impact on feed efficiency. The results suggest that the HRFI pigs in the study of Mani *et al*. [2] are suffering from an increased basal level of inflammation and heightened immune activity which was in contrast to the current study where a differential immune response was only exhibited following a challenge. The results from the current study and from the literature [2, 31, 32] indicate that the lower inflammatory cytokine gene expression in response to a challenge in the LRFI group may be an energy saving mechanism, but despite this may possibly have an increased ability to respond to infection.

Regulation of cytokine signalling occurs at multiple levels, including limiting the ability of cytokines to initiate an immune response. The SOCS proteins are involved in the control of cytokine signalling [12]. The results from this study are therefore surprising as there is an interaction between RFI and the LPS challenge for *SOCS3* with HRFI pigs having lower expression following the challenge. Primarily *SOCS3* regulates *STAT3* through the gp130 receptor which forms part of the receptor for the *IL-6* complex so it is to be expected that increased SOCS3 expression in the HRFI group would lead to a reduction in *IL-6* [33], but this was not observed. One possible explanation is that the level of *SOCS3* in the LRFI group is sufficient to reduce the levels of IL-6 whereas the HRFI group despite having increased *SOCS3* are unable to regulate the expression of *IL-6*. This ties in with the previously developed hypothesis that LRFI pigs have a more efficient and co-ordinated immune response. Alternatively in studies with mice without *SOCS3*, *IL-6* becomes anti-inflammatory suggesting a function of *SOCS3* is to maintain the pro-inflammatory response of *IL-6* [34]. This could explain the results from this study where *SOCS3* expression does not lower the expression of *IL-6*.

## Conclusion

In summary, in the basal non-infected state, LRFI pigs had increased *lactobacilli*, suggesting a possible improvement in gut health. However, in the basal state RFI groups did not differ in the measured innate immune responses with no difference in the gene expression of the measured mucin genes, TLRs, tight junction proteins, cytokines or SOCS genes in either the ileum or colon. The lower expression of *IL-1*, *IL-6*, *IL-8*, *IL-10* and *TNF-α*. and *SOCS3* following an *ex vivo* LPS challenge suggests that an energy saving mechanism possibly exists in the intestine in the more feed efficient animals which impacts on their immune response to infection.
